# The Hypothalamic Arcuate Nucleus Dopaminergic Neurons: More Than Just Prolactin Secretion

**DOI:** 10.1210/endocr/bqaf025

**Published:** 2025-02-07

**Authors:** Luis Wei Cheng Lim, Christopher Thomas Egnot, Panagiotis Papaioannou, Siew Hoong Yip

**Affiliations:** Centre for Neuroendocrinology, Department of Anatomy, University of Otago, Dunedin 9016, New Zealand; Centre for Neuroendocrinology, Department of Anatomy, University of Otago, Dunedin 9016, New Zealand; Centre for Neuroendocrinology, Department of Anatomy, University of Otago, Dunedin 9016, New Zealand; Centre for Neuroendocrinology, Department of Anatomy, University of Otago, Dunedin 9016, New Zealand; Maurice Wilkins Centre for Molecular Biodiscovery, Auckland 1010, New Zealand

**Keywords:** A12 neurons, dopamine, prolactin, neuronal heterogeneity

## Abstract

The hypothalamic arcuate nucleus dopaminergic (A12) neurons are well known for their central role in regulating prolactin secretion through a sophisticated negative feedback loop. In this canonical pathway, prolactin stimulates A12 neurons to release dopamine, which suppresses further prolactin release from lactotrophs in the anterior pituitary. However, a collective of recent and past evidence strongly implies that the A12 neurons are far more dynamic and multifaceted than previously appreciated. This minireview discusses the developmental trajectory of A12 neurons, from prenatal origins to postnatal maturation, highlighting their diversity and heterogeneity. Beyond their well-characterized role in prolactin regulation, the A12 neurons contribute to a broader array of hypothalamic functions, including autoregulation, metabolism, and growth. By shedding light on these underexplored roles, this review outlines the expansive significance of A12 neurons as more than mere gatekeepers of prolactin secretion, positioning them as versatile players in endocrine and metabolic homeostasis.

The mediobasal hypothalamus of the brain is the core region of central regulation of neuroendocrine hormone secretion from the pituitary gland. It houses sets of neuroendocrine neural circuits which are intertwined to receive and redistribute cues from central and peripheral organs. In turn, they influence other neuroendocrine systems to achieve homeostasis. Prolactin, discovered by Dr. Oscar Riddle in the early 1930s, is a polypeptide hormone that is well known for its role in promoting milk synthesis during lactation (1). Over the last 90 years, understanding of its physiological role has expanded enormously with multiple central and peripheral target organs. The pleiotropic nature of this hormone is reflected by its sophisticated dopaminergic regulatory system, allowing the rise and fall of its levels at the right times to appropriately regulate a range of physiological functions (2). There are approximately 16 clusters of catecholaminergic neurons throughout the rodent midbrain and forebrain, at which 8 clusters are dopaminergic neurons, termed A8 to A16 (3). Among these, 3 neuroendocrine dopaminergic neuron groups regulate prolactin secretion. The 2 rostrally located groups are the tuberohypophyseal (THDA) and periventricular hypophyseal (PHDA) dopaminergic neurons (4). The A12 is a group of dopaminergic neurons residing in the tuberal region of the hypothalamus (arcuate nucleus). The majority of these A12 neurons project to the infundibular structure (median eminence) and they are thus known as tuberoinfundibular dopaminergic (TIDA) neurons. While it is widely accepted that A12 dopaminergic neurons are TIDA neurons, especially those located in the dorsomedial region of the arcuate nucleus, this minireview challenges this perspective by presenting evidence that A12 neurons exhibit diverse molecular profiles and do not exclusively project to the median eminence but also to other brain regions. This becomes more apparent with more recent evidence showing that the A12 dopamine neurons regulate growth, body weight, and metabolism (5, 6). This minireview will describe the role of the A12 neuronal system in regulating prolactin secretion and beyond. It will specifically focus on the developmental aspects of these neurons, both prenatally and postnatally, which contribute to the diversity of their phenotypes and, consequently, their functions.

## Development of A12 Neurons

### Prenatal Development of A12 Neurons

**Figure 1.**
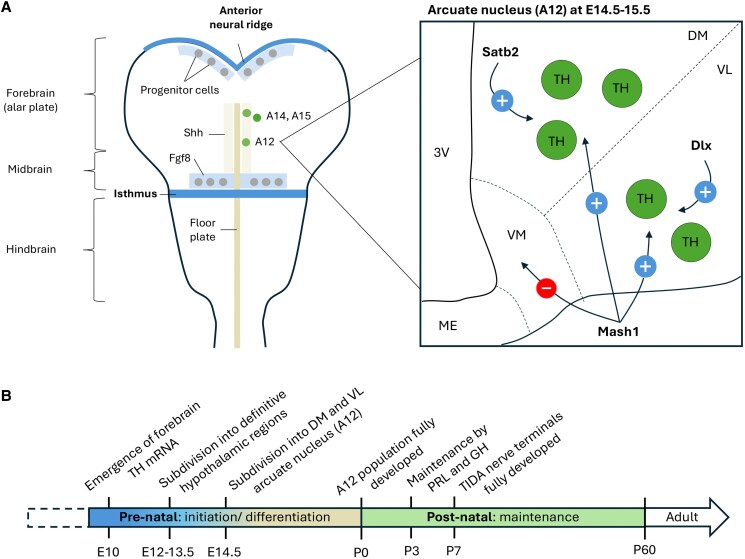
Prenatal and postnatal development of A12 dopaminergic neurons. (A) Schematic dorsal view (left panel) of a developing rodent brain, highlighting dopamine progenitor cells located adjacent to the anterior neural ridge and isthmus. The formation of dopaminergic progenitor cells into definite hypothalamic population (A12, A14, A15) along the anteroposterior axis is dependent on a balance of Shh signaling from the midline floor plate and Fgf8 signaling from the isthmus and anterior neural ridge. The coronal view of the arcuate nucleus (A12; right panel) illustrates the influence of Satb2 and Dlx genes in driving the initiation and differentiation of A12 TH (tyrosine hydroxylase; green) neurons into dorsomedial (DM) and ventrolateral (VL) subdivisions, respectively. The Mash1 gene promotes the development of A12 TH neurons while suppressing ventromedial (VM) TH cell formation. (B) Timeline showing the stages of TH neuron development from prenatal day E10 to postnatal day P60. Abbreviations: 3V, third ventricle; ME, median eminence.

Dopaminergic clusters throughout midbrain and forebrain originate from 2 groups of progenitor cells lining the ventral neural tube: (i) rostral to the isthmus structure (a boundary between the midbrain and hindbrain); and (ii) a small region in the anterior neural ridge (7) (Fig. 1A). While the exact anatomical fate of these progenitor cells remains unknown, several well-recognized factors involved in the formation and differentiation of dopaminergic neurons include sonic hedgehog (Shh), fibroblast growth factor 8 (Fgf8), Nkx2.1, Dlx homeobox, and the proneural transcription factor Mash1 as reviewed by Hynes and Rosenthal in 1999 (8).

The formation of progenitor cells of these dopaminergic neurons in the ventral neuroepithelium lining along anteroposterior axis relies on a balance of Shh signaling from the floor plate and Fgf8 signaling molecule of the isthmus and anterior neural ridge (Fig. 1A). In vitro studies have demonstrated that Shh-producing floor plate explants induce ectopic differentiation of dopamine neurons dorsal to the progenitor cells, while neutralizing Shh with antibodies prevents the development of these neurons (7). Similarly, blocking Fgf8 signaling from cephalic neural plate explants results in the absence of tyrosine hydroxylase (TH)-positive cells (rate-limiting enzyme of dopamine synthesis). These signals not only induce the formation of progenitor cells but also influence them to produce specific combinations of transcription factors, establishing distinct progenitor domains along the anteroposterior and dorsoventral axes of the neural plate. These domains eventually differentiate into specific clusters of dopaminergic neurons (8).

While the development of midbrain dopaminergic neurons (A8-A10) has been extensively studied (see review by Hergaty et al, 2013) (9), less is known about forebrain dopaminergic neurons. The emergence of TH mRNA in the forebrain was first observed at embryonic day (E) 10 in mouse (10), localized to the lateral wall of the forebrain (alar plate). By E12, these alar plate neurons progressively shift into more definitive hypothalamic positions, giving rise to the A12 (arcuate nucleus), A14 (periventricular nucleus), and A15 (preoptic nucleus) dopaminergic neuron clusters. This progression is supported by immunohistochemical staining of TH showing the presence of TH-immunoreactive neurons in the hypothalamic area as early as E13.5 in rats (11) (Fig. 1B).

The adult A12 population is subdivided into dorsomedial and ventrolateral regions. These neurons are first detected at E14.5, where TH-expressing cells emerge in the ventrolateral region, followed by the dorsomedial region of the arcuate nucleus at E15.5 (Figure 1A). Concurrently, the transcription factor Satb2 (special AT-rich sequence-binding protein 2) is detected in a significant proportion of A12 neurons, highlighting its importance in the development of the A12 population (12, 13). In fact, knocking out Satb2 in the central nervous system using nestin-cre/Satb2-flox transgenic mice results in a reduction of TH-expressing cells in the arcuate nucleus from E14.5 compared to wild-type embryos. Interestingly, this reduction is restricted to the dorsomedial region at E14.5, while the ventrolateral region shows a notable TH reduction only after postnatal day 14 (13). This suggests that Satb2 drives the initiation and differentiation of dorsomedial A12 dopamine neurons, including TIDA neurons, while it is essential for maintaining ventrolateral TH neurons.

Other critical genes, such as Nkx2.1, Dlx homeobox, and Mash1, are also essential for the development and function of A12 neurons. Lineage tracing of A12 neurons using transgenic approaches with Cre-recombinase driven by either Nkx2.1 or Dlx (coupled with GFP reporters) revealed that each gene is expressed in only half of the TH neurons in the A12 population at postnatal day 0. Although their overlapping expression was not determined, knocking out Dlx genes led to a significant reduction in TH neurons, specifically in the ventrolateral region of the A12 population (14). However, the role of Nkx2.1 on the A12 neurons remain elusive. The Mash1 gene is critical not only for neurogenesis but also for apoptosis in the A12 population. A study by McNay et al demonstrated that knocking out both alleles of Mash1 in a mouse model resulted in hypoplasia of the arcuate nucleus and failure of TH neuron development (15). Interestingly, knocking out a single allele of Mash1 led to an opposite effect, significantly increasing TH neurons in the ventromedial region of the arcuate nucleus—a region where TH neurons are largely absent in wild-type adult mice. This indicates that while Mash1 is essential for the generation of A12 TH neurons, it also plays a role in shaping the final subdivisions of these neurons observed in adulthood (Fig. 1A).

### Postnatal Development of A12 Neurons

While most A12 neurons are generated during prenatal development, the development of TIDA nerve terminal projections to the median eminence occurs only after postnatal day 7 (16). The maintenance of the A12 neurons requires a balance of hormones, particularly anterior pituitary hormones such as prolactin and growth hormone (GH). Mutations in the pituitary transcription factor-1 (Pit-1) in Snell dwarf mice or its upstream transcription factor, Prophet of Pituitary transcription factor-1 (Prop-1), in Ames dwarf mice result in undetectable levels of these hormones. These mutations are associated with a remarkable regression in the size of the dopaminergic neuron population, specifically the A12 population, during postnatal development. However, there is a temporal dichotomy in the postnatal stages at which the loss occurs between the 2 mouse models (16, 17). In Snell mice, A12 neuron regression is evident as early as postnatal day 7, whereas in Ames mice, it does not occur until day 30 (16).

A high dose of daily heterologous ovine prolactin (50 µg) replacement via intraperitoneal injection into both Ames and Snell dwarf mice, starting between postnatal day 3 and day 42, has been shown to effectively preserve the A12 TH population (16, 18) comparable to wild-type mice (19). It is worth noting that while a lower dose of ovine prolactin (5 µg) can also prevent regression of the A12 population, it does not achieve the same level of rescue as the high-dose treatment. Furthermore, when a high dose of homologous recombinant mouse prolactin treatment (50 µg) is administered, specific to prolactin receptor, the rescued population size is smaller than with heterologous ovine prolactin. This suggests the involvement of an additional factor, potentially GH, in driving this effect. Indeed, daily heterologous porcine GH (50 µg) replacement from postnatal day 3 to day 43 in both Snell and Ames mice partially preserves the A12 population (16, 20). Prolactin and GH are members of a family of polypeptide hormones that share structural similarities and biological activities, and they could activate not only their respective but also each other receptors. Moreover, the A12 neurons do express GH receptors (21). This observation may explain the dose-dependent effect of prolactin replacement and differences between heterologous and homologous prolactin treatment on the preservation of the A12 population. While a high dose of heterologous ovine prolactin could activate both prolactin and GH receptors on A12 neurons (21) leading to full rescue, a low dose of heterologous ovine prolactin or a high dose of homologous recombinant prolactin may only activate prolactin receptors alone, hence unable to fully rescue the regression. Whether GH and prolactin acts synergistically or independently to maintain A12 neurons during postnatal development remains unknown. Together, these studies highlight the vital roles of prolactin and GH in maintaining A12 neurons from postnatal development through to adulthood.

## Heterogeneity of A12 Neurons

Over the last few decades, differential protein and gene expression among A12 neurons has been well documented. This heterogeneity manifests in many forms, including differences in receptor expression, neurotransmitter handling, and firing patterns. Such diversity among A12 neurons is most likely established during the complex yet distinct developmental pathways described in earlier sections, where neurons differentiate into subtypes based on specific genetic programs. In fact, single-cell sequencing using Drop-seq to catalog cell types from the arcuate nucleus of both male and female mice revealed 6 clusters of dopaminergic subtypes (22). Among them, 2 clusters are identified as TIDA neurons by the authors based on the criteria that they express both prolactin receptors and dopamine transporters. Between these 2 clusters of TIDA neurons, only one expresses somatostatin mRNA. While the function of somatostatin in TIDA neurons remains unknown, this finding underscores the diversity of the A12 neuronal population. In the following subsections, the differential molecular, morphological, and electrical profiles among these neurons will be highlighted.

### Molecular Profile Diversity

As described in earlier section, dopaminergic neurons in the arcuate nucleus begin differentiating into distinct anatomical divisions as early as embryonic day 14.5. At this stage, TH neurons in the ventrolateral region express growth hormone–releasing hormone (GHRH) and are regulated independently from the dorsomedially located TH neurons (23, 24). The dorsomedial A12 neurons are primarily TIDA neurons; however, some of them express GABA which are not hypophysiotropic, hence do not regulate prolactin secretion (25), and some are non-dopaminergic mono-enzymatic TH neurons that produce L-DOPA (26, 27). Numerous other studies also demonstrated subsets of A12 neurons differentially express (i) receptor proteins, including those for prolactin (28), dopamine (29), growth hormone (5), ghrelin and leptin (30) receptors; (ii) membrane transporters, such as dopamine transporters (5, 31, 32), and vesicular GABA transporters (25, 33); and (iii) intracellular proteins and transcription factors, including Rho A, period circadian proteins (PER1/2), and CLOCK (30, 34). Such diversity in the molecular profiles reflects a myriad of physiological roles of the A12 neurons in addition to regulation of prolactin secretion. Some of these roles will be discussed in later sections.

### Morphological Profile Diversity

Classic Golgi impregnation and more advanced techniques, such as biocytin cell filling or viral vector transduction of protein markers, have revealed diverse morphologies within the dorsomedial A12 population, including TIDA neurons. Their cell bodies may be fusiform or round, with some neurons displaying dendritic spines while others do not. Their neuronal processes emanate either from the cell bodies or dendrites (29, 35-37). While the significance of these morphological differences remains unclear, they further add to the evidence of heterogeneity of the A12 population.

### Electrical and Calcium Activity Profile Diversity

In addition to molecular and morphological diversity, the electrical activity of A12 neurons, particularly the TIDA neurons is also heterogeneous, at least in mice, and can be categorized into silent, bursting, irregular, and fast-regular firing patterns (38-41). Similarly, in vivo calcium (Ca^2+^) imaging using GRIN lens technology of A12 neurons in both male and female mice revealed 4 different clusters of calcium profile based on the duration and frequency of Ca^2+^ peaks (42). The significance of this differential electrical profile and dopamine output will be discussed in a later section.

The diversity of the above 3 profiles is further amplified under different physiological conditions, with one of the most remarkable and well-studied examples occurring during lactation, when the neurons undergo adaptive plasticity. The lactation-associated neurotransmitter switch from dopamine to enkephalin in A12 neurons was first reported in the early 1990s in rats and mice (43, 44). This finding was later supported by more recent investigations using a transgenic mouse model (45), which intriguingly highlighted that only a subpopulation of dorsomedial A12 neurons, including the TIDA neurons, express enkephalin. Enkephalin plays an essential role in promoting prolactin secretion from lactotrophs (45). Furthermore, morphological analyses of A12 neurons demonstrated variations in dendritic spine density among neurons in virgin female rats, with a higher density observed in many, though not all, during lactation (37). This observation suggests increased synaptic input to A12 neurons, perhaps the TIDA neurons during lactation, which may account for the shift from slow to tonic oscillatory firing frequency patterns, ultimately leading to reduced dopamine output and elevated prolactin (46). Collectively, these adaptive changes in a subset of A12 neurons, the TIDA neurons are shown to promote prolactin release, which is critical for lactation. However, the functions of other subsets of A12 neurons during lactation remain largely unknown.

Overall, these findings clearly highlight the heterogeneity of A12 neurons, as evidenced by differences in multiple aspects within the same population of neurons. While the connections between genetic composition, neurochemical content, and firing activities remain difficult to determine, the observed heterogeneity of A12 neurons strongly suggests that they may have multifaceted functions. It also emphasizes that TIDA neurons are a subpopulation of A12 neurons that project to and release dopamine at the median eminence to regulate prolactin secretion. Therefore, the term *TIDA* should not be loosely linked to all A12 neurons. In the following sections, we will discuss the canonical role of A12 neurons (the TIDA neurons) in prolactin regulation, followed by emerging and potential roles beyond prolactin secretion.

## TIDA Neurons Regulate Prolactin Release

The adult TIDA neurons are well-established as key regulators of prolactin secretion from lactotrophs in the anterior pituitary gland. They achieve this by tonically suppressing spontaneous prolactin secretion from the lactotrophs. Treatment with monosodium glutamate in rat neonates results in a reduction in the number of TH cells in the arcuate nucleus, but not in other dopaminergic populations, leads to an increase in prolactin levels in adulthood (27). The release of dopamine from TIDA neurons is regulated through multiple mechanisms, including their own spontaneous activity as well as prolactin stimulation via a short-loop negative feedback.

### Spontaneous Activity of TIDA Neurons

TIDA neurons have been shown to exhibit intrinsic pacemaking activity, in the form of highly rhythmic oscillations, with the peaks of these oscillations characterized by depolarization and bursts of action potentials. This activity is proposed to arise from the interplay between T-type calcium (Ca^2+^) channels and type 3 small conductance Ca^2+^-activated potassium channels (SK3) (47). The generation of this pacemaking rhythmic oscillatory pattern depends on Na^+^ channels, where application of tetrodotoxin completely abolishes the TIDA oscillation (48). Whereas the frequency of these spontaneous rhythmic properties is intrinsically regulated by the transient receptor potential channel 5 (TRPC5) and is independent of synaptic origin. Blockade of glutamate and GABA receptors did not alter the rhythmicity frequency of the TIDA neurons (49, 50).

In male and female mice, electrophysiological recordings revealed that not all TIDA neurons exhibit bursting/oscillatory activity; some display fast, nonbursting, irregular rhythms while others remain silent (39-42, 51). Imaging intracellular Ca^2+^ transients revealed similar patterns in an ex vivo setup from both male and female mice. These recorded Ca^2+^ activities are classified into long-lasting Ca^2+^ peaks, short-duration peaks with high or low oscillation frequencies, and silent states (40, 42). In male rats, such diverse patterns of TIDA firing activities are lacking, with the majority of the neurons uniformly displaying the bursting pattern. This species dichotomy in TIDA spontaneous firing activity appears to be due to the presence of gap junctions, at least in male rats, which phase-lock the neurons into a synchronous and hence uniform network (40, 48). Meanwhile, the spontaneous activity patterns of TIDA neurons in female rats remain underexplored in the literature (52).

The spontaneous activity patterns of TIDA neurons play a key role in regulating their dopamine release dynamics and consequently, prolactin secretion. Optogenetic or electrical manipulation of the TIDA neurons oscillation frequency (53) and action potential frequency (51) in male mice, significantly alters dopamine output at the median eminence ex vivo and corresponds to the changes in blood prolactin levels in vivo. Similarly, disrupting the activity profiles of A12 neurons (including TIDA neurons) in female mice, by knocking out their expression of TRPC5, results in significant changes in blood prolactin levels (50), indicating altered dopamine output from these neurons. In male rats, the robust network synchronization of TIDA neurons, is essential to maintain their slow rhythmic oscillatory firing patterns (48), a characteristic hallmark of efficient dopamine release (40). Disrupting this synchronization using pharmacological gap junction blockers diminishes these oscillatory characteristics (48), thereby failing to maintain low blood prolactin levels (40).

Although gap junction expression is lacking in mouse TIDA neurons (40), a recent in vivo Ca^2+^ imaging study demonstrated that these neurons can still achieve episodes of synchronized activity (42). These synchronization episodes effectively suppress prolactin secretion and correlate with, and are possibly driven by, an increase in the number of TIDA neurons concurrently displaying long-duration Ca^2+^ peaks. Prolactin is suggested as the likely factor mediating episodic synchronization in vivo. This idea is supported by ex vivo observations where episodic synchronization is largely absent. However, the application of prolactin results in the spatiotemporal reorganization of long-duration Ca^2^⁺ peak TIDA neurons into a synchronized network (42). Interestingly, in vivo Ca^2+^ imaging revealed sex differences in the spontaneous activity of TIDA neurons, with male mice exhibiting significantly higher numbers of TIDA neurons with long-duration Ca^2+^ peaks compared to female mice. Given that long-duration Ca^2+^ peaks correspond to TIDA network synchronization and efficient prolactin suppression, their differential prevalence between the sexes would explain the sexual dimorphism in blood prolactin levels, with male mice having significantly lower prolactin levels compared to female mice. The same study also highlighted the importance of the connectivity (topology) of TIDA neurons in forming functional networks. Using functional segregation and integration computational models, their topologies can be categorized into varying strengths of functional networks, each corresponding to different efficacies in suppressing prolactin secretion (42). The authors further revealed sexual differences in the in vivo topological organization of TIDA neurons, with male mice exhibiting significantly stronger network topologies, which corresponded to lower blood prolactin levels.

Together, these intricate intrinsic activities of TIDA neurons ultimately maintain homeostatic low levels of prolactin within the endocrine system. These findings also highlight sexual dimorphism in long-duration Ca^2+^ peaks and network topologies in vivo, which may be attributed to the extra afferent synaptic inputs that male mice TIDA neurons receive from different brain regions compared to female mice (54). It is worth noting that due to the limited literature on the activity of TIDA neurons in species beyond rats and mice, the regulation of prolactin secretion by these neurons in other species remains largely unknown.

### Prolactin-Induced Activity of TIDA Neurons

In addition to the spontaneous intrinsic activity, TIDA neurons can be induced by prolactin to synthesize and release dopamine. This is crucial for forming a prolactin-mediated negative feedback loop, whereby prolactin stimulates TIDA neurons to release dopamine, which in turn inhibits further prolactin secretion; for review refer to Phillipps et al (2). The spontaneously released prolactin from the lactotrophs gains access into the brain through either active transport (55) or prolactin-induced prolactin release from the choroid plexus (56). However, the exact mode of transport remains controversial. Once in the brain, prolactin regulates a myriad of neural circuits, including stimulating dopamine output from the TIDA neurons (57, 58) which predominantly express the long forms of prolactin receptors (28). Studies using acute brain slices from mice and rats have demonstrated that the application of prolactin results in a near-instantaneous increase in the firing rate of TIDA neurons (38, 39, 49, 50), which corresponds to transient elevations of dopamine at the median eminence, as measured by amperometry (39).

**Figure 2.**
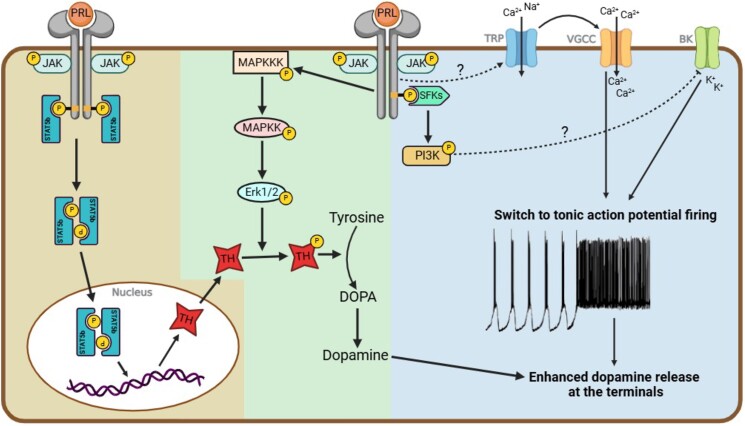
Prolactin-induced dopamine release from TIDA neurons. Schematic depicting the action of prolactin on downstream intracellular signaling pathways and the dynamics of voltage-gated channels. Activation of prolactin receptors leads to the phosphorylation of both the pSTAT5 and MAPK/ERK pathways, resulting in the transcription of the TH gene and phosphorylation of TH, respectively, which in turn increases dopamine biosynthesis. Additionally, prolactin receptor activation independently activates the PI3K and TRP channels, leading to the dynamic opening and closing of voltage-gated calcium channels (VGCCs) and voltage-gated potassium channels (BK). This causes a switch in the firing pattern of TIDA neurons, enhancing dopamine release at the nerve terminals.

At the individual cellular level, the binding of prolactin to its receptors activates the Src family of kinases (SFKs), leading to the phosphorylation of the phosphatidylinositol 3-kinase (PI3K) pathway. PI3K plays a crucial role in prolactin-induced action potential discharge (high-voltage component of inward current) of TIDA neurons. This process is mediated through L-type voltage-gated Ca^2^⁺ channels and voltage-gated potassium channels. In male rat brain slices, inhibition of PI3K with wortmannin prevents TIDA neurons from reaching the action potential firing threshold, suggesting that PI3K may modulate the opening of both these channel types (49) (Fig. 2). The same study in male rats also demonstrated another channel activated by prolactin, independent of PI3K, namely the TRPC channel. These channels allow a slow influx of Na⁺ and Ca^2^⁺ into the cell, resulting in sub-threshold depolarization (low-voltage inward current). The application of TRPC blockers, such as 2-APB, leads to the inhibition of the low-voltage but not the high-voltage components of prolactin-induced inward currents, confirming the existence of 2 distinct pathways mediating prolactin actions on TIDA firing activity (49). While the involvement of PI3K remains unclear in mice, the deletion of TRPC in TRPC5 deficient female mice completely prevents the prolactin-evoked excitation of TIDA neurons (50). It should be noted, however, that there are at least 4 distinct firing patterns of A12 neurons in mice. Therefore, the electrical recordings of arcuate nucleus TH-positive neurons in the mouse studies may not exclusively reflect bona fide TIDA neurons. Together, activation of PI3K and/ or TRPC by prolactin promote further depolarization, resulting in a switch from phasic to tonic firing of action potentials in the TIDA neurons, enhancing dopamine release bouts (Fig. 2). At the cellular network level, *in vivo* administration of exogenous prolactin to female mouse shifted them to a male-like state, with an increased number of TIDA neurons displaying long Ca^2+^ peaks and enhanced activity synchronization, accompanied by a corresponding suppression of endogenous prolactin secretion (42).

In addition to promoting dopamine release, prolactin may evoke changes in intracellular signaling pathways in TIDA neurons to enhance the biosynthesis of dopamine, thereby maintaining the optimal capacity of its output (Fig. 2). This can be achieved in 2 ways: (i) an acute increase in tyrosine hydroxylase (TH) activity via phosphorylation of this rate-limiting enzyme; and (ii) a chronic upregulation of TH gene transcription. The mitogen-activated protein kinase (MAPK/Erk) pathway is thought to mediate, at least in part, the rapid prolactin-induced site-specific phosphorylation of TH, particularly at serine-40 (Fig. 2). Prolactin treatment in neonatal rat hypothalamic cell cultures has been shown to increase phosphorylation of Erk1/2, accompanied by a rise in phosphorylated TH within 60 minutes and a “delayed” rise in TH mRNA levels at 120 minutes (59). Additionally, prolactin activates the signal transducer and activator of transcription factor (STAT5) pathway, exclusively the STAT5b isoform in the brain (60). STAT5b-deficient male and female mice exhibit an overall reduction in TH mRNA levels and a decreased number of TH-expressing cells in the arcuate nucleus, correlated with elevated prolactin levels compared to their wild-type counterparts (61). This suggests the involvement of the STAT5 pathway in TH gene transcription, linking it to prolactin's regulatory effects on dopamine biosynthesis (Fig. 2).

**Figure 3.**
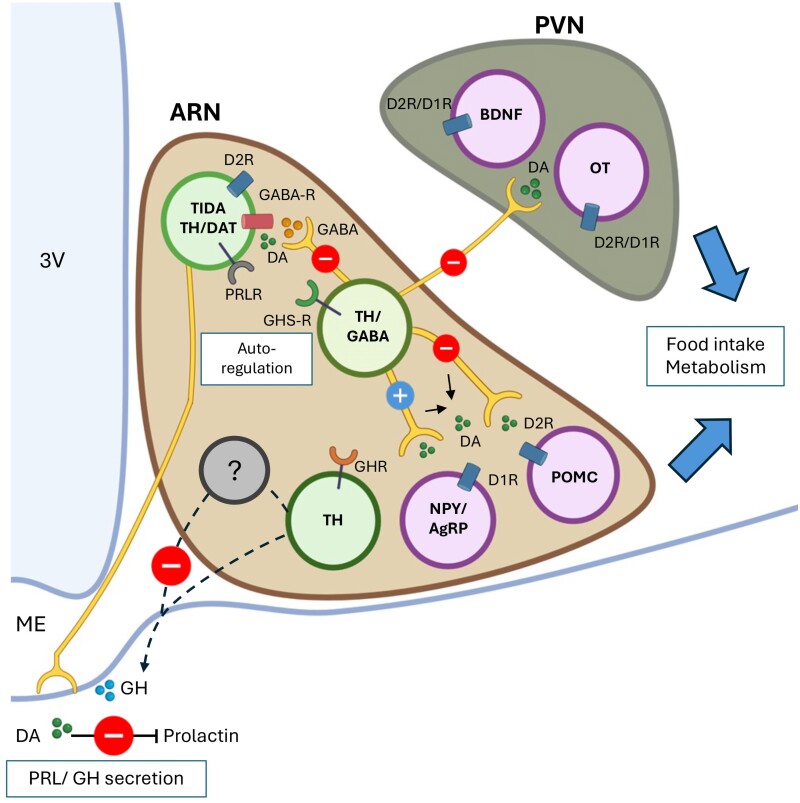
Functional role of subpopulations of A12 neurons. Schematic illustrating 3 subpopulations of A12 TH neurons expressing either GABA, dopamine transporter (DAT), or growth hormone receptor (GHR), in the arcuate nucleus (ARN). The TIDA subpopulation of A12 neurons that express prolactin receptors (PRLR) and DAT project to the median eminence (ME) and are responsible for the prolactin negative feedback mechanism. The ghrelin sensitive TH/GABA subpopulations may serve 2 functions: (i) Autoregulation of TIDA neurons; and (ii) driving orexigenic effects by innervating dopamine-sensitive metabolic regulatory neurons. The latter occurs via the inhibition of pro-opiomelanocortin (POMC) neurons, and the stimulation of neuropeptide-Y/agouti-related protein (NPY/AgRP) neurons located locally in the ARN. The TH/GABA neurons also project to the paraventricular nucleus (PVN) and may inhibit both brain-derived neurotrophic factor (BDNF) and oxytocin (OT) neurons. A12 TH neurons expressing GHR, but not co-expressing DAT, are required for the inhibition of growth hormone (GH) secretion; however, whether this occurs through direct or indirect action via interneurons remains unknown (dashed line). Abbreviations: 3V, third ventricle; GHS-R, ghrelin receptor; D1R, dopamine receptor type 1; D2R, dopamine receptor type 2.

Collectively, the activation of the prolactin receptor drives an intricate network of intracellular signaling pathways. These pathways increase the transcription and activation of TH, as well as firing activity, subsequently enhancing dopamine release at the median eminence. Dopamine is then transported via the hypophyseal portal vein to the anterior pituitary gland, where it binds to dopamine type-2 receptors (D2R) on lactotrophs. This results in the suppression of further prolactin secretion, completing the short-loop negative feedback mechanism by which prolactin regulates its own release from the anterior pituitary gland (Fig. 3).

## Emerging Role of TIDA Neurons

A broader role of A12 neurons, apart from regulating prolactin secretion, is perhaps not surprising considering the vast number of afferent inputs these neurons receive. Trans-synaptic retrograde tracing specifically from A12 neurons revealed that they receive inputs from more than 50 different brain regions, both intra- and extrahypothalamic (54). In addition, A12 neurons express a variety of receptors that are sensitive to brain and peripheral signals. A comprehensive review of inputs to A12 neurons can be found in several other reviews (47, 62) and a book chapter (63). It is therefore reasonable to assume that these integrated signals are translated not only to regulate prolactin secretion but also to mediate other functions that have been less studied. While hyperprolactinemia was observed in mice with prolactin receptor deletion from all forebrain neurons, including the entire A12 population, conditional deletion of prolactin receptors from all GABAergic neurons, including the arcuate nucleus TH/GABA subpopulation, did not alter circulating prolactin levels in these mice (25). This rules out the role of TH/GABA subpopulation of A12 neurons in regulation of prolactin secretion. Further supporting this, the authors show that there is a lack of colocalization of vGAT and TH nerve terminals at the external zone of the median eminence. Such findings strongly suggest that at least this GABAergic subpopulation of A12 neurons, some of which located in the dorsomedial region of arcuate nucleus, has a distinct functional role beyond prolactin regulation. In addition, this study also highlights that identifying prolactin regulating TIDA neurons based on merely anatomical location being dorsomedial located A12 neurons, was overly simplistic. In fact, both anterograde and retrograde neuronal tracing studies demonstrated that not all dorsomedial arcuate nucleus located A12 neurons project to the median eminence. Some form local axon collaterals, while others project to brain regions outside the arcuate nucleus (4, 6). Therefore, this final section of the minireview aims to highlight a few emerging roles of A12 neurons, particularly in the regulation of body weight, growth hormone secretion, and autoregulation among A12 neurons themselves. These findings align with a perspective by Grattan and Akopian, who described A12 neurons as “multitasking” neurons (64).

### Regulation of Body Weight and Metabolism

Dopamine signaling has long been implicated in regulating appetite by integrating reward-related processes with homeostatic pathways (for review see Baik et al, 2021) (65). Most previous studies have focused on the A8 to A10 dopaminergic populations (66), whereas the A12 population remains less explored. Early evidence demonstrated that lesioning the arcuate nucleus of male rats via stereotaxic injection of colchicine led to hypothalamic obesity, accompanied by alterations in food intake, body weight, and subcutaneous fat (67). Given that the arcuate nucleus also houses the well known anorexigenic pro-opiomelanocortin (POMC) and orexigenic neuropeptide-Y/agouti-related protein (NPY/AgRP) neurons, the potential role of A12 neurons was initially overlooked. Interestingly, diet-induced obesity in male mice has been shown to increase phosphorylation of TH in the hypothalamus, suggesting enhanced activity of hypothalamic dopaminergic neurons (30). Furthermore, Satb2 gene deletion, which impedes the development of A12 neurons (as described earlier), also results in reduced body weight and metabolic rate in mice (13). Notably, Satb2 is not expressed in either POMC or AgRP neurons. Therefore, these findings suggest that A12 neurons may play a role in body weight and metabolic regulation, if not in hypothalamic obesity.

Aligning with this notion, there is compelling evidence that A12 neurons, particularly the GABAergic subpopulation (25, 33), are involved in energy homeostasis (6). In male mice, food deprivation for 24 hours activates TH neurons in the arcuate nucleus, as evidenced by increased cFos expression and higher spontaneous neuronal firing rates, which are critical for driving food intake after fasting. Consistently, using G-protein-coupled receptor-activation-based sensors to monitor dopamine release in the arcuate nucleus of overnight-fasted male and female mice revealed food-induced dopamine release (68). Moreover, in vivo optogenetic photoactivation of arcuate TH neurons resulted in increased food intake (6). Collectively, these findings strongly suggest the role of A12 neurons in body weight, food intake, and metabolic regulations, at least in mice. However, it is important to note that these manipulations also included the ventrolateral A12 TH neurons that co-express growth hormone–releasing hormone (23, 69), which may influence body size and weight in the studied mice. Interestingly, cFos analyses of A12 neurons in male mice conducted by another research group yielded opposing results, showing decreased neuronal activity during fasting (70). The underlying cause of these conflicting findings remains unknown, further complicating our understanding of the role of A12 neurons in energy balance.

It becomes increasingly clear that A12 neurons are responsive to hunger, as they express ghrelin receptors (30). The direct action of ghrelin on A12 neurons was demonstrated by ghrelin-induced depolarization, even in the presence of TTX or a cocktail of GABA/glutamate receptor antagonists (6). Conversely, selective silencing of arcuate TH neurons using tetanus toxin analog and Cre-lox technology resulted in reduced body weight gain in male mice. These findings indicate that A12 neurons, can integrate and control food intake, at least in the male mice. However, whether this function is independent or involves local interactions with other arcuate neurons, such as POMC and NPY/AgRP neurons or neurons located elsewhere in the brain, remains unknown.

Recent studies have uncovered projections of A12 neurons to brain regions involved in metabolic regulation (6, 71). Using intersectional targeting approaches with double transgenic TH-Cre; VGAT-Flp male and female mice to selectively transduce AAV-protein markers into arcuate TH/GABA neurons, it was reported that these neurons not only form local axon collaterals within the arcuate nucleus, as shown previously in mice and rats (36, 37), but also project to distal brain regions including the paraventricular, ventromedial, and dorsolateral hypothalamic nuclei, key centers for food intake and metabolic regulation (Fig. 3). Sexual differences in A12 neurons innervation pattern were not evaluated.

Within the arcuate nucleus, TH/GABA subpopulation of A12 neurons are likely to target dopamine receptor-expressing POMC and NPY/AgRP neurons (68, 72). While NPY/AgRP neurons predominantly express the excitatory dopamine receptor type 1 (D1R), POMC neurons express the inhibitory dopamine receptor type 2 (D2R) (72) (Fig. 3). This aligns with findings showing inhibition of POMC neurons upon application of the dopamine agonist SKF38393 or optogenetic stimulation of TIDA neurons (6). While dopamine inhibits POMC neurons in both male and female mice, heat map perforated patch-clamp analysis suggests that female POMC neurons are more sensitive to dopamine, as a greater proportion of these neurons were inhibited in females compared to males upon *ex vivo* dopamine application (72). In contrast, the interaction between A12 neurons and NPY/AgRP neurons remains controversial. Although pharmacological activation of dopamine receptors excites NPY/AgRP neurons, optogenetic activation of A12 neurons failed to elicit excitatory effects on these orexigenic neurons in male mice (6). This suggests that A12 neurons may not directly innervate NPY/AgRP neurons, or at least not via synaptic contact, but rather through bulk transmission (Fig. 3). However, a subsequent study contradicted this by showing direct synaptic contacts between A12 neurons and NPY/AgRP neurons (73). Using rabies virus-based trans-synaptic tracing with poly-A nuclear transcriptomics to map synaptic inputs, the study confirmed synaptic inputs between these 2 neural circuits. Further investigation is required to validate this interaction and clarify the role of A12 neurons in regulating NPY/AgRP neurons. Notably, majority of the A12 neurons, including the TH/GABA subpopulation are responsive to prolactin (25), hence prolactin activation of this subpopulation of A12 neurons could account for hyperprolactinemic link to obesity (74).

Another metabolic center innervated by TH/GABA A12 neurons is the paraventricular nucleus (PVN) (6) (Fig. 3). In male mice, photo-stimulation of ChIEF-transduced TH somata in the arcuate nucleus or their nerve terminals in the PVN evoked inhibitory postsynaptic currents in the majority of PVN neurons. The degree of opto-induced inhibition was reduced by application of D2 receptor antagonists and further attenuated by addition of the GABA receptor antagonist bicuculline, suggesting that release of both dopamine and GABA from A12 neurons are the key transmitters that suppress local PVN neurons. The exact target cells within the PVN remain largely unknown but may include key metabolic regulators such as melanocortin receptor 4-expressing neurons, brain-derived neurotrophic factor (BDNF), oxytocin, and calcitonin neurons. Immunohistochemical studies in rats revealed that a significant number of BDNF and oxytocin neurons in the PVN express both D1R and D2R (75, 76) implying that they may be the target of A12 neurons innervation (Fig. 3).

In summary, current evidence suggests that A12 neurons, specifically the TH/GABA neurons, drive orexigenic effects by interacting with metabolic regulatory neurons, particularly those in the arcuate nucleus and PVN. Further studies are needed to identify the specific PVN neurons targeted by these A12 neurons. Additionally, more studies are needed to confirm similar role of A12 neurons in female models, especially considering the maternal plasticity of TIDA neurons during lactation, when they switch from producing dopamine to enkephalin (45). It is worth exploring whether such plasticity is part of maternal adaptive changes in respond to metabolic challenges during this stage of reproduction.

### Autoregulation of A12 Neurons

In addition to the local actions of A12 neurons on metabolic regulatory neurons within the arcuate nucleus, they also exert autocrine actions among themselves. Stagkourakis and colleagues provided convincing evidence that A12 neurons are involved in autoregulation via D2R in male rats (Fig. 3). By increasing dopaminergic transmission using the selective D2R agonist quinpirole or by blocking dopamine reuptake, the oscillation frequency of A12 neuron firing activity was shown to slow down (77). A subsequent study demonstrated that, despite the ability of exogenous GABA_B_ receptor agonists to inhibit A12 neurons, blockade of the receptor alone failed to alter their spontaneous oscillation frequency. This finding shows a lack of endogenous GABA_B_ receptor activation effect (78) and therefore suggests that suppressive action of dopamine relies on presynaptic inhibitory GABAergic tone. Although the exact sources of dopamine and GABA could not be pinpointed in these studies. The ex vivo setup, which primarily consisted of the A12 population, led the authors to postulate that the likely source is local release of dopamine from the dendrites or axon collaterals of A12 neurons themselves—an autoregulatory feature also observed in dopaminergic populations in the midbrain (79). This hypothesis is further supported by morphological studies revealing multiple modes of interaction among A12 neurons, including dendro-dendritic, axo-dendritic, dendro-somatic, and axo-axonic putative contacts in female rats (37). Overall, A12 neurons play a role in autoregulation, at least in the rats, as a means of immediate homeostatic tuning, which ultimately influences their primary functions in regulation of prolactin secretion and perhaps other roles outline in this section.

### Regulation of Growth Hormone Secretion

GH pulsatile secretion is regulated by negative feedback loops, in which GH feeds back to GHRH and somatostatin (Sst)-expressing neurons (80). Recent evidence implicates A12 neurons in the regulation of GH pulsatile secretion (5). First, A12 neurons are responsive to GH, as demonstrated by acute porcine GH injections inducing pSTAT5 responses in the majority of TH neurons within the arcuate nucleus. Second, deletion of GH receptors (GH-R) from all TH neurons in male mice resulted in increased GH pulsatile secretion and corresponding somatic growth compared to control wild-type mice (5). While the effect of receptor deletion on GH pulsatile secretion has not been directly demonstrated in female mice, these mice show higher somatic growth compared to sex-matched wild-type controls, suggesting increased GH pulsatile secretion (5). In addition, it remains unclear which subpopulation of A12 neurons is responsive to GH and whether they regulate GH secretion from the pituitary directly or indirectly. In order to identify specific subpopulation of A12 neurons that is involved in the regulation of growth hormone secretion, the authors employed Cre-lox transgenic approach to delete GH-R: TH-Cre (all catecholaminergic neurons), DAT-Cre (dopaminergic neurons), DBH-Cre (adrenergic/noradrenergic neurons), and Nestin-Cre (whole-brain neurons as control that includes all 3 subpopulations). As mentioned above, deletion of GH-R from all TH neurons resulted in increased somatic growth compared to control wild-type mice. However, deletion of the receptor specifically from DAT/TH neurons or DBH/TH neurons has no effect on the somatic growth in both sexes. This indicates that although the DAT- and DBH-expressing TIDA subpopulation is responsive to GH, it is not involved in feedback regulation of GH. Although the specific A12 subpopulation responsible for this regulation remains to be identified, the DAT/TH-expressing A12 neurons are not involved in mediating the negative feedback regulation of GH secretion (Fig. 3). As described earlier, there is a cluster of arcuate nucleus TH cells that express Sst mRNA, as identified by single-cell Drop-seq (22) and thus potential candidate. Nevertheless, using Sst-IRES-Cre mice crossed with *td-Tomato* reporter mice, the authors ruled out their involvement by showing a lack of colocalization between Sst-*td-Tomato* immunostaining and arcuate nucleus TH cells (5). Alternatively, the authors explored possible involvement of the GHRH/TH subpopulation in the arcuate nucleus (23, 69) by testing their response to GH. However, the number of such neurons is low, and only a few of them respond to GH. Collectively, this study clearly demonstrates another role for A12 neurons, in controlling GH pulsatile secretion and, consequently, body growth in both male and female mice. Nevertheless, the exact subpopulation of A12 neurons mediating this action, as well as whether they regulate GH secretion directly or indirectly via local or distal interneurons (Fig. 3), remains to be determined.

## Conclusion

In conclusion, while A12 neurons have long been recognized for their role in feedback regulation of prolactin, emerging evidence highlights their multifaceted functions of the neuroendocrine system. This review highlights the complex development of A12 neurons from prenatal to postnatal stages, potentially leading to heterogeneous subpopulations with distinct molecular signatures and electrical properties. However, the connections between these characteristics are not yet well understood and remain challenging to elucidate. Such heterogeneity in the A12 population suggests a broader spectrum of physiological functions beyond negative feedback regulation of prolactin, therefore challenging the conventional understanding of A12 neurons as mainly TIDA neurons and opening new avenues for exploring their roles in broader physiological processes. Despite these advances, significant questions remain regarding the precise correlation between specific subpopulations and their functions. Future research should focus on bridging the gap between molecular and electrophysiological profiles to unravel the functional diversity of A12 neurons and their roles in neuroendocrine regulation. Specifically, pinpointing the overlap between molecular expression patterns and electrophysiological behaviors, to accurately map specific A12 subpopulations to their corresponding functions. This endeavor will not only deepen our understanding of A12 neuron heterogeneity but may also uncover novel mechanisms underlying neuroendocrine regulation. These potential insights will be critical to draw closer to the understanding of the physiological significance of A12 neurons in health and disease.

## Data Availability

Data sharing is not applicable to this article as no data sets were generated or analyzed during the current study.

## Abbreviations

A12 neurons: hypothalamic arcuate nucleus dopaminergic neurons
AgRP: agouti-related protein
BDNF: brain-derived neurotrophic factor
D1R: dopamine receptor type 1
D2R: dopamine receptor type 2
E#: embryonic day #
Fgf8: fibroblast growth factor 8
GH: growth hormone
GHRH: growth hormone–releasing hormone
NPY: neuropeptide-Y
POMC: pro-opiomelanocortin
PVN: paraventricular nucleus
Satb2: special AT-rich sequence-binding protein 2
Shh: sonic hedgehog
Sst: somatostatin
TH: tyrosine hydroxylase
TIDA: tuberoinfundibular dopaminergic
TRPC5: transient receptor potential channel 5

## References

[1] Smith MS . Anterior pituitary hormones: development of a bioassay leading to the discovery of prolactin. Am J Physiol Endocrinol Metab. 2004;287(5):E813‐E814. 15475508 10.1152/classicessays.00022.2004

[2] Phillipps HR, Yip SH, Grattan DR. Patterns of prolactin secretion. Mol Cell Endocrinol. 2019;502:110679. 31843563 10.1016/j.mce.2019.110679

[3] Björklund A, Dunnett SB. Dopamine neuron systems in the brain: an update. Trends Neurosci 2007;30(5):194-202. 17408759 10.1016/j.tins.2007.03.006

[4] Kawano H, Daikoku S. Functional topography of the rat hypothalamic dopamine neuron systems: retrograde tracing and immunohistochemical study. J Comp Neurol. 1987;265(2):242‐253. 2891732 10.1002/cne.902650208

[5] Wasinski F, Pedroso JAB, Dos Santos WO, et al Tyrosine hydroxylase neurons regulate growth hormone secretion via short-loop negative feedback. J Neurosci. 2020;40(22):4309‐4322. 32317389 10.1523/JNEUROSCI.2531-19.2020 PMC7252485

[6] Zhang X, van den Pol AN. Hypothalamic arcuate nucleus tyrosine hydroxylase neurons play orexigenic role in energy homeostasis. Nat Neurosci. 2016;19(10):1341‐1347. 27548245 10.1038/nn.4372 PMC6402046

[7] Ye W, Shimamura K, Rubenstein JL, Hynes MA, Rosenthal A. FGF and Shh signals control dopaminergic and serotonergic cell fate in the anterior neural plate. Cell. 1998;93(5):755‐766. 9630220 10.1016/s0092-8674(00)81437-3

[8] Hynes M, Rosenthal A. Specification of dopaminergic and serotonergic neurons in the vertebrate CNS. Curr Opin Neurobiol. 1999;9(1):26‐36. 10072377 10.1016/s0959-4388(99)80004-x

[9] Hegarty SV, Sullivan AM, O’Keeffe GW. Midbrain dopaminergic neurons: a review of the molecular circuitry that regulates their development. Dev Biol. 2013;379(2):123‐138. 23603197 10.1016/j.ydbio.2013.04.014

[10] Marín F, Herrero MT, Vyas S, Puelles L. Ontogeny of tyrosine hydroxylase mRNA expression in mid- and forebrain: neuromeric pattern and novel positive regions. Dev Dyn. 2005;234(3):709‐717. 15973733 10.1002/dvdy.20467

[11] Daikoku S, Kawano H, Okamura Y, Tokuzen M, Nagatsu I. Ontogenesis of immunoreactive tyrosine hydroxylase-containing neurons in rat hypothalamus. Brain Res. 1986;393(1):85‐98. 2873873 10.1016/0165-3806(86)90068-4

[12] Huang Y, Song NN, Lan W, et al Expression of transcription factor Satb2 in adult mouse brain. Anat Rec (Hoboken). 2013;296(3):452‐461. 23386513 10.1002/ar.22656

[13] Zhang Q, Zhang L, Huang Y, et al Satb2 regulates the development of dopaminergic neurons in the arcuate nucleus by Dlx1. Cell Death Dis. 2021;12(10):879. 34564702 10.1038/s41419-021-04175-9 PMC8464595

[14] Yee CL, Wang Y, Anderson S, Ekker M, Rubenstein JL. Arcuate nucleus expression of NKX2.1 and DLX and lineages expressing these transcription factors in neuropeptide Y(+), proopiomelanocortin(+), and tyrosine hydroxylase(+) neurons in neonatal and adult mice. J Comp Neurol. 2009;517(1):37‐50. 19711380 10.1002/cne.22132 PMC3021751

[15] McNay DE, Pelling M, Claxton S, Guillemot F, Ang SL. Mash1 is required for generic and subtype differentiation of hypothalamic neuroendocrine cells. Mol Endocrinol. 2006;20(7):1623‐1632. 16469766 10.1210/me.2005-0518

[16] Phelps CJ . Postnatal regression of hypothalamic dopaminergic neurons in prolactin-deficient Snell dwarf mice. Endocrinology. 2004;145(12):5656‐5664. 15345680 10.1210/en.2004-0931

[17] Phelps CJ, Carlson SW, Vaccarella MY, Felten SY. Developmental assessment of hypothalamic tuberoinfundibular dopamine in prolactin-deficient dwarf mice. Endocrinology. 1993;132(6):2715‐2722. 8504771 10.1210/endo.132.6.8504771

[18] Romero MI, Phelps CJ. Prolactin replacement during development prevents the dopaminergic deficit in hypothalamic arcuate nucleus in prolactin-deficient Ames dwarf mice. Endocrinology. 1993;133(4):1860‐1870. 8104778 10.1210/endo.133.4.8104778

[19] Khodr CE, Hurley DL, Phelps CJ. Prolactin induces tuberoinfundibular dopaminergic neurone differentiation in Snell dwarf mice if administered beginning at 3 days of age. J Neuroendocrinol. 2009;21(6):558‐567. 19500226 10.1111/j.1365-2826.2009.01869.x PMC2695862

[20] Khodr CE, Clark S, Bokov AF, et al Early postnatal administration of growth hormone increases tuberoinfundibular dopaminergic neuron numbers in Ames dwarf mice. Endocrinology. 2010;151(7):3277‐3285. 20463054 10.1210/en.2009-1482 PMC2903943

[21] Wasinski F, Chaves FM, Pedroso JAB, et al Growth hormone receptor in dopaminergic neurones regulates stress-induced prolactin release in male mice. J Neuroendocrinol. 2021;33(3):e12957. 33769619 10.1111/jne.12957 PMC9670090

[22] Campbell JN, Macosko EZ, Fenselau H, et al A molecular census of arcuate hypothalamus and median eminence cell types. Nat Neurosci. 2017;20(3):484‐496. 28166221 10.1038/nn.4495 PMC5323293

[23] Zoli M, Agnati LF, Tinner B, Steinbusch HW, Fuxe K. Distribution of dopamine-immunoreactive neurons and their relationships to transmitter and hypothalamic hormone-immunoreactive neuronal systems in the rat mediobasal hypothalamus. A morphometric and microdensitometric analysis. J Chem Neuroanat. 1993;6(5):293‐310. 7506039 10.1016/0891-0618(93)90034-2

[24] Hurley DL, Bartke A, Wagner TE, Wee BE, Phelps CJ. Increased hypothalamic somatostatin expression in mice transgenic for bovine or human GH. J Neuroendocrinol. 1994;6(5):539‐548. 7827624 10.1111/j.1365-2826.1994.tb00617.x

[25] Brown RS, Kokay IC, Phillipps HR, et al Conditional deletion of the prolactin receptor reveals functional subpopulations of dopamine neurons in the arcuate nucleus of the hypothalamus. J Neurosci. 2016;36(35):9173‐9185. 27581458 10.1523/JNEUROSCI.1471-16.2016 PMC6601914

[26] Ugrumov M, Melnikova V, Ershov P, Balan I, Calas A. Tyrosine hydroxylase- and/or aromatic L-amino acid decarboxylase-expressing neurons in the rat arcuate nucleus: ontogenesis and functional significance. Psychoneuroendocrinology. 2002;27(5):533‐548. 11965353 10.1016/s0306-4530(01)00091-9

[27] Bodnár I, Göõz P, Okamura H, et al Effect of neonatal treatment with monosodium glutamate on dopaminergic and L-DOPA-ergic neurons of the medial basal hypothalamus and on prolactin and MSH secretion of rats. Brain Res Bull. 2001;55(6):767‐774. 11595361 10.1016/s0361-9230(01)00584-6

[28] Kokay IC, Wyatt A, Phillipps HR, et al Analysis of prolactin receptor expression in the murine brain using a novel prolactin receptor reporter mouse. J Neuroendocrinol. 2018;30(9):e12634. 30040149 10.1111/jne.12634

[29] Romero-Fernandez W, Borroto-Escuela DO, Vargas-Barroso V, et al Dopamine D1 and D2 receptor immunoreactivities in the arcuate-median eminence complex and their link to the tubero-infundibular dopamine neurons. Eur J Histochem. 2014;58(3):2400. 25308843 10.4081/ejh.2014.2400 PMC4194391

[30] Skov LJ, Ratner C, Hansen NW, et al Rhoa in tyrosine hydroxylase neurones regulates food intake and body weight via altered sensitivity to peripheral hormones. J Neuroendocrinol. 2019;31(7):e12761. 31237372 10.1111/jne.12761

[31] Ciliax BJ, Heilman C, Demchyshyn LL, et al The dopamine transporter: immunochemical characterization and localization in brain. J Neurosci. 1995;15(3):1714‐1723. 7534339 10.1523/JNEUROSCI.15-03-01714.1995 PMC6578165

[32] Yip SH, York J, Hyland B, Bunn SJ, Grattan DR. Incomplete concordance of dopamine transporter Cre (DAT(IREScre))-mediated recombination and tyrosine hydroxylase immunoreactivity in the mouse forebrain. J Chem Neuroanat. 2018;90:40‐48. 29217488 10.1016/j.jchemneu.2017.12.002

[33] Negishi K, Payant MA, Schumacker KS, et al Distributions of hypothalamic neuron populations coexpressing tyrosine hydroxylase and the vesicular GABA transporter in the mouse. J Comp Neurol. 2020;528(11):1833‐1855. 31950494 10.1002/cne.24857 PMC7993550

[34] Sellix MT, Egli M, Poletini MO, et al Anatomical and functional characterization of clock gene expression in neuroendocrine dopaminergic neurons. Am J Physiol Regul Integr Comp Physiol. 2006;290(5):R1309‐R1323. 16373438 10.1152/ajpregu.00555.2005 PMC1457054

[35] Chan-Palay V, Zaborszky L, Kohler C, Goldstein M, Palay SL. Distribution of tyrosine-hydroxylase-immunoreactive neurons in the hypothalamus of rats. J Comp Neurol. 1984;227(4):467‐496. 6147362 10.1002/cne.902270403

[36] Herde MK, Iremonger KJ, Constantin S, Herbison AE. GnRH neurons elaborate a long-range projection with shared axonal and dendritic functions. J Neurosci. 2013;33(31):12689‐12697. 23904605 10.1523/JNEUROSCI.0579-13.2013 PMC6618539

[37] Yip SH, Araujo-Lopes R, Szawka RE, et al Morphological plasticity of the tuberoinfundibular dopaminergic neurones in the rat during the oestrous cycle and lactation. J Neuroendocrinol. 2020;32(11):e12884. 32662600 10.1111/jne.12884

[38] Brown RS, Piet R, Herbison AE, Grattan DR. Differential actions of prolactin on electrical activity and intracellular signal transduction in hypothalamic neurons. Endocrinology. 2012;153(5):2375‐2384. 22416085 10.1210/en.2011-2005

[39] Romano N, Yip SH, Hodson DJ, et al Plasticity of hypothalamic dopamine neurons during lactation results in dissociation of electrical activity and release. J Neurosci. 2013;33(10):4424‐4433. 23467359 10.1523/JNEUROSCI.4415-12.2013 PMC6704969

[40] Stagkourakis S, Pérez CT, Hellysaz A, Ammari R, Broberger C. Network oscillation rules imposed by species-specific electrical coupling. Elife. 2018;7:e33144. 29722649 10.7554/eLife.33144 PMC5933921

[41] Zhang X, van den Pol AN. Dopamine/tyrosine hydroxylase neurons of the hypothalamic arcuate nucleus release GABA, communicate with dopaminergic and other arcuate neurons, and respond to dynorphin, met-enkephalin, and oxytocin. J Neurosci. 2015;35(45):14966‐14982. 26558770 10.1523/JNEUROSCI.0293-15.2015 PMC4642233

[42] Cherepanov S, Heitzmann L, Fontanaud P, et al Prolactin blood concentration relies on the scalability of the TIDA neurons’ network efficiency in vivo. iScience. 2024;27(6):109876. 38799572 10.1016/j.isci.2024.109876 PMC11126972

[43] Merchenthaler I . Induction of enkephalin in tuberoinfundibular dopaminergic neurons during lactation. Endocrinology. 1993;133(6):2645‐2651. 7694844 10.1210/endo.133.6.7694844

[44] Ciofi P, Crowley WR, Pillez A, Schmued LL, Tramu G, Mazzuca M. Plasticity in expression of immunoreactivity for neuropeptide Y, enkephalins and neurotensin in the hypothalamic tubero-infundibular dopaminergic system during lactation in mice. J Neuroendocrinol. 1993;5(6):599‐602. 8680430 10.1111/j.1365-2826.1993.tb00528.x

[45] Yip SH, Romanò N, Gustafson P, et al Elevated prolactin during pregnancy drives a phenotypic switch in mouse hypothalamic dopaminergic neurons. Cell Rep. 2019;26(7):1787‐1799.e5. 30759390 10.1016/j.celrep.2019.01.067

[46] Thörn Pérez C, Ferraris J, van Lunteren JA, Hellysaz A, Iglesias MJ, Broberger C. Adaptive resetting of tuberoinfundibular dopamine (TIDA) network activity during lactation in mice. J Neurosci. 2020;40(16):3203‐3216. 32209609 10.1523/JNEUROSCI.1553-18.2020 PMC7159885

[47] Qi-Lytle X, Sayers S, Wagner EJ. Current review of the function and regulation of tuberoinfundibular dopamine neurons. Int J Mol Sci. 2023;25(1):110. 38203281 10.3390/ijms25010110 PMC10778701

[48] Lyons DJ, Horjales-Araujo E, Broberger C. Synchronized network oscillations in rat tuberoinfundibular dopamine neurons: switch to tonic discharge by thyrotropin-releasing hormone. Neuron. 2010;65(2):217‐229. 20152128 10.1016/j.neuron.2009.12.024

[49] Lyons DJ, Hellysaz A, Broberger C. Prolactin regulates tuberoinfundibular dopamine neuron discharge pattern: novel feedback control mechanisms in the lactotrophic axis. J Neurosci. 2012;32(23):8074‐8083. 22674282 10.1523/JNEUROSCI.0129-12.2012 PMC6620951

[50] Blum T, Moreno-Pérez A, Pyrski M, et al Trpc5 deficiency causes hypoprolactinemia and altered function of oscillatory dopamine neurons in the arcuate nucleus. Proc Natl Acad Sci U S A. 2019;116(30):15236‐15243. 31285329 10.1073/pnas.1905705116 PMC6660783

[51] Stagkourakis S, Dunevall J, Taleat Z, Ewing AG, Broberger C. Dopamine release dynamics in the tuberoinfundibular dopamine system. J Neurosci. 2019;39(21):4009‐4022. 30782976 10.1523/JNEUROSCI.2339-18.2019 PMC6529860

[52] Briffaud V, Williams P, Courty J, Broberger C. Excitation of tuberoinfundibular dopamine neurons by oxytocin: crosstalk in the control of lactation. J Neurosci. 2015;35(10):4229‐4237. 25762669 10.1523/JNEUROSCI.2633-14.2015 PMC6605300

[53] Stagkourakis S, Smiley KO, Williams P, et al A neuro-hormonal circuit for paternal behavior controlled by a hypothalamic network oscillation. Cell. 2020;182(4):960‐975.e15. 32763155 10.1016/j.cell.2020.07.007 PMC7445434

[54] Esteves FF, Matias D, Mendes AR, Lacoste B, Lima SQ. Sexually dimorphic neuronal inputs to the neuroendocrine dopaminergic system governing prolactin release. J Neuroendocrinol. 2019;31(10):e12781. 31419363 10.1111/jne.12781 PMC6851580

[55] Brown RS, Wyatt AK, Herbison RE, et al Prolactin transport into mouse brain is independent of prolactin receptor. FASEB J. 2016;30(2):1002‐1010. 26567005 10.1096/fj.15-276519

[56] Costa-Brito AR, Quintela T, Gonçalves I, et al The choroid plexus is an alternative source of prolactin to the rat brain. Mol Neurobiol. 2021;58(4):1846‐1858. 33409838 10.1007/s12035-020-02267-9

[57] Bole-Feysot C, Goffin V, Edery M, Binart N, Kelly PA. Prolactin (PRL) and its receptor: actions, signal transduction pathways and phenotypes observed in PRL receptor knockout mice. Endocr Rev. 1998;19(3):225‐268. 9626554 10.1210/edrv.19.3.0334

[58] Grattan DR . Behavioural significance of prolactin signalling in the central nervous system during pregnancy and lactation. Reproduction. 2002;123(4):497‐506. 11914112 10.1530/rep.0.1230497

[59] Ma FY, Grattan DR, Goffin V, Bunn SJ. Prolactin-regulated tyrosine hydroxylase activity and messenger ribonucleic acid expression in mediobasal hypothalamic cultures: the differential role of specific protein kinases. Endocrinology. 2005;146(1):93‐102. 15388649 10.1210/en.2004-0800

[60] Yip SH, Eguchi R, Grattan DR, Bunn SJ. Prolactin signalling in the mouse hypothalamus is primarily mediated by signal transducer and activator of transcription factor 5b but not 5a. J Neuroendocrinol. 2012;24(12):1484‐1491. 22775396 10.1111/j.1365-2826.2012.02357.x

[61] Grattan DR, Xu J, McLachlan MJ, et al Feedback regulation of PRL secretion is mediated by the transcription factor, signal transducer, and activator of transcription 5b. Endocrinology. 2001;142(9):3935‐3940. 11517172 10.1210/endo.142.9.8385

[62] Lyons DJ, Broberger C. TIDAL WAVES: network mechanisms in the neuroendocrine control of prolactin release. Front Neuroendocrinol. 2014;35(4):420‐438. 24561279 10.1016/j.yfrne.2014.02.001

[63] Szawka RE, Bunn SJ, Le Tissier P, Yip SH, Grattan DR. Lactation and the control of the prolactin secretion. In: Brunton PJ, Grattan DR, eds. Neuroendocrine Regulation of Mammalian Pregnancy and Lactation. Springer International Publishing; 2024:181‐221.

[64] Grattan DR, Akopian AN. Oscillating from neurosecretion to multitasking dopamine neurons. Cell Rep. 2016;15(4):681‐682. 27119847 10.1016/j.celrep.2016.04.013 PMC4890962

[65] Baik JH . Dopaminergic control of the feeding circuit. Endocrinol Metab (Seoul). 2021;36(2):229‐239. 33820393 10.3803/EnM.2021.979 PMC8090468

[66] Palmiter RD . Is dopamine a physiologically relevant mediator of feeding behavior? Trends Neurosci. 2007;30(8):375‐381. 17604133 10.1016/j.tins.2007.06.004

[67] Choi S, Sparks R, Clay M, Dallman MF. Rats with hypothalamic obesity are insensitive to central leptin injections. Endocrinology. 1999;140(10):4426‐4433. 10499495 10.1210/endo.140.10.7064

[68] Zhang Q, Tang Q, Purohit NM, et al Food-induced dopamine signaling in AgRP neurons promotes feeding. Cell Rep. 2022;41(9):111718. 36450244 10.1016/j.celrep.2022.111718 PMC9753708

[69] Phelps CJ, Romero MI, Hurley DL. Growth hormone-releasing hormone-producing and dopaminergic neurones in the mouse arcuate nucleus are independently regulated populations. J Neuroendocrinol. 2003;15(3):280‐288. 12588517 10.1046/j.1365-2826.2003.01009.x

[70] Kubota T, Fukushima A, Hagiwara H, et al Short-term fasting decreases excitatory synaptic inputs to ventromedial tuberoinfundibular dopaminergic neurons and attenuates their activity in male mice. Neurosci Lett. 2018;671:70‐75. 29438798 10.1016/j.neulet.2018.02.017

[71] Mittal S, Arenkiel BR, Lyons-Warren AM. Arcuate dopaminergic/GABAergic neurons project within the hypothalamus and to the median eminence. J Neurophysiol. 2024;132(3):943‐952. 39108212 10.1152/jn.00086.2024 PMC11427037

[72] Gaziano I, Corneliussen S, Biglari N, et al Dopamine-inhibited POMCDrd2+ neurons in the ARC acutely regulate feeding and body temperature. JCI Insight. 2022;7(21):e162753. 36345942 10.1172/jci.insight.162753 PMC9675440

[73] Webster AN, Becker JJ, Li C, et al Molecular connectomics reveals a glucagon-like peptide 1-sensitive neural circuit for satiety. Nat Metab. 2024;6(12):2354‐2373. 39627618 10.1038/s42255-024-01168-8 PMC12186539

[74] Lopez-Vicchi F, Ladyman SR, Ornstein AM, et al Chronic high prolactin levels impact on gene expression at discrete hypothalamic nuclei involved in food intake. FASEB J. 2020;34(3):3902‐3914. 31944423 10.1096/fj.201902357R

[75] Zhou L, Zhang Y, Lian H, Li Y, Wang Z. Colocalization of dopamine receptors in BDNF-expressing peptidergic neurons in the paraventricular nucleus of rats. J Chem Neuroanat. 2020;106:101794. 32315740 10.1016/j.jchemneu.2020.101794

[76] Ran X, Yang Y, Meng Y, et al Distribution of D(1) and D(2) receptor- immunoreactive neurons in the paraventricular nucleus of the hypothalamus in the rat. J Chem Neuroanat. 2019;98:97‐103. 31018158 10.1016/j.jchemneu.2019.04.002

[77] Stagkourakis S, Kim H, Lyons DJ, Broberger C. Dopamine autoreceptor regulation of a hypothalamic dopaminergic network. Cell Rep. 2016;15(4):735‐747. 27149844 10.1016/j.celrep.2016.03.062 PMC4850423

[78] Ammari R, Broberger C. Pre- and post-synaptic modulation by GABAB receptors of rat neuroendocrine dopamine neurones. J Neuroendocrinol. 2020;32(11):e12881. 32803906 10.1111/jne.12881

[79] Ford CP, Gantz SC, Phillips PE, Williams JT. Control of extracellular dopamine at dendrite and axon terminals. J Neurosci. 2010;30(20):6975‐6983. 20484639 10.1523/JNEUROSCI.1020-10.2010 PMC2883253

[80] Steyn FJ, Huang L, Ngo ST, et al Development of a method for the determination of pulsatile growth hormone secretion in mice. Endocrinology. 2011;152(8):3165‐3171. 21586549 10.1210/en.2011-0253

